# Beyond Skin Deep: Patient Insights Into Cosmetic Procedures and Their Risks in Madinah, Saudi Arabia

**DOI:** 10.7759/cureus.74399

**Published:** 2024-11-25

**Authors:** Ibrahim H Babikir, Taif S Aloufi, Raghad K Alyahyawi, Fatima R Alarabi, Bochra A Abdelkerim, Rahaf M Alraddadi, Mohammed F Al ayoubi, Mohammed A Elmuttalut

**Affiliations:** 1 Department of Microbiology, Al-Rayan National College of Medicine, Madinah, SAU; 2 Department of Clinical Sciences, Al-Rayan National College of Medicine, Madinah, SAU; 3 Department of Cosmetics, Neo-Derma Clinic, Madinah, SAU; 4 Department of Community Medicine, Al-Rayan National College of Medicine, Madinah, SAU; 5 Department of Community Medicine, College of Medicine, University of Sinnar, Sinnar, SDN

**Keywords:** botox injections, bruising, cosmetic awareness, cosmetic complications, cosmetic dermatology, cosmetic procedure, facelift procedures, lip filler, rash, swelling

## Abstract

Background

The demand for cosmetic procedures has increased significantly due to their positive effects on self-satisfaction. However, individuals often underestimate the potential complications associated with these procedures, driven by a strong desire for transformation. Therefore, this study aimed to evaluate the Madinah community's awareness of cosmetic procedures and plastic surgery, as well as to assess the risks, complications, and clinical outcomes associated with these procedures.

Materials and methods

A non-probability consecutive sampling method was employed to select study participants, including every patient who met the inclusion criteria until the target sample size was reached, between March and June 2023. An intervener-based questionnaire survey was conducted to assess participant’s understanding of the severity and associated risks of cosmetic procedures and plastic surgery. The minimum required sample size was determined to be 82 using OpenEpi version 3 software. Data were analyzed using t-tests, chi-square tests, and analysis of variance (ANOVA) as appropriate.

Results

The study included 82 participants, with 48.80% aged between 18 and 30 years and only 3.7% of them were male. The majority were of Saudi nationality (86.60%). While most participants (82.9%) were aware of cosmetic procedures and their risks, 28% lacked knowledge about potential complications, highlighting a notable knowledge gap. Some of the participants reported underlying health conditions such as obesity (8.5%), diabetes (3.7%), asthma (3.7%), and hypertension (2.4%), along with family histories of hereditary diseases (37.8%) and allergies (15.9%). The most common complications were facial swelling (63.41%), bruising (51.22%), rashes (30.49%), and subcutaneous blood pooling (19.51%). Less frequent complications included infections (15.85%) and reduced facial expressions (9.76%).

Conclusion

The study revealed that most participants demonstrated an overall awareness of the potential complications associated with cosmetic procedures. The commonly recognized complications of cosmetic facial procedures included swelling, bruising, rash, subcutaneous blood pooling, and infection. However, no specific factors related to the planning of facelift procedures were identified.

## Introduction

The concept of beauty has been fundamental since the beginning of human existence [1]. In recent years, the demand for cosmetic procedures has largely increased due to their positive impacts on self-esteem [1, 2]. Notably, many individuals overlook the possible complications associated with these procedures in their eagerness for transformation [2]. While most adverse effects from cosmetic procedures are temporary and range from mild to moderate, there are also significant risks, such as infection and reduced facial expressions caused by subcutaneous blood pooling [1, 3]. Therefore, it is crucial for patients to be aware of the signs and symptoms of complications and be well-informed about the potential risks before deciding to undergo any cosmetic procedure [4].

Individuals and civilizations throughout history have consistently sought to enhance their attractiveness. For instance, ancient Egyptians employed a variety of substances - including animal oils, salt, alabaster, and sour milk - to improve the aesthetics of their skin [1]. Modern cosmetic procedures, such as filler and Botox injections, illustrate the shift from traditional natural skin remedies to scientifically advanced treatments designed for aesthetic enhancement. Filler injections use hyaluronic acid, a natural skin substance that maintains moisture and volume, to add fullness, smooth wrinkles, and improve texture, especially in areas like the cheeks, lips, and mouth. Meanwhile, Botox involves a protein derived from *Clostridium botulinum* (botulinum toxin), which, when injected in small doses, temporarily relaxes facial muscles to reduce wrinkles and fine lines around the eyes and forehead. These procedures highlight how cosmetic science has evolved to provide targeted, effective solutions for skin rejuvenation [1-3].

Globally, the number of minimally invasive facial aesthetic procedures performed annually continues to rise [3, 4]. In 2017 alone, over 8.5 million nonsurgical injection procedures were conducted worldwide, marking an increase of nearly 850,000 since 2015 [5]. In recent decades, cosmetic procedures have gained immense popularity, driven by a desire for a youthful appearance despite the natural aging process [6, 7]. The preference for cosmetic procedures has been significantly amplified by exposure through various media channels, including the internet, television, and social media, leading to increased public awareness [8, 9]. However, research has explored both the popularity of common cosmetic procedures and the associated complications. In their pursuit of beauty, individuals often overlook the potential complications, which can arise as early, transient, delayed, or even prolonged issues [10-12].

Among the most common complications of cosmetic procedures are bruising, changes in skin pigmentation, burns, infections, scarring, swelling, and the development of "frozen" facial expressions. Additional complications may include seroma formation and nerve damage, which can lead to sensory loss. In severe cases, skin breakdown or disfigurement may occur [13, 14]. Consequently, these complications may significantly affect the patient’s emotional and physical well-being, as well as their overall quality of life. However, there is hope, as prompt intervention can mitigate these adverse outcomes. Many of these complications, if managed early and effectively, can prevent long-term damage and disfigurement [10].

Recently, the Kingdom of Saudi Arabia, particularly in Madinah, has experienced a surge in the availability of cosmetic procedures due to advancements in medical technology. Despite the numerous clinics and centers offering a variety of services, materials, and tools, there is a concerning lack of health awareness regarding these procedures. This lack of awareness has contributed to an increase in complications resulting from cosmetic procedures in Madinah.

Therefore, this study aims to evaluate the participant’s awareness of cosmetic procedures and plastic surgery, as well as to assess the associated risks, complications, and clinical outcomes of these procedures.

## Materials and methods

Study design and data collection

This study was conducted as a descriptive, facility-based cross-sectional survey from March to June 2023 in Madinah, Saudi Arabia. The primary objective was to assess awareness levels regarding cosmetic procedures, plastic surgery, and their associated risks. Data were gathered using a structured, pretested, and validated interviewer-administered questionnaire, designed to evaluate participants' knowledge of potential risks and clinical outcomes of cosmetic procedures.

Inclusion and exclusion criteria

*Inclusion criteria*: Patients, 18 years old or above, attending Neo-Derma Private Clinic in Madinah, who consented to participate by signing an ethical consent form, were included in the study.

*Exclusion criteria*: Participants younger than 18 years, those with incomplete or missing data, or those unwilling to participate or sign the consent form were excluded from the study.

Sampling technique and sample size

The sample size was calculated using OpenEpi, Version 3, with a total of 82 participants needed to reach an 80% power level to detect a 5% difference at a significance level of α = 0.05, assuming a 10% attrition rate. A non-probability consecutive sampling method was used, selecting participants who met the inclusion criteria until the target sample size was achieved.

Study area, location, and duration

The study was conducted at the Neo-Derma Clinic in Madinah, Saudi Arabia, over four months, from March to June 2023.

Statistical analysis

Data entry, management, and analysis were performed using IBM SPSS Statistics (Version 25.0, IBM Corp., Armonk, NY). The descriptive data were analyzed and summary statistics, including frequencies, percentages, were calculated to characterize the sample and illustrate key sociodemographic and clinical variables. Categorical variables such as age group, gender, nationality, education level, job, monthly income, medical history, and awareness of cosmetic procedures were analyzed using frequency distributions and percentages. Descriptive data were presented as frequency tables and graphs. The chi-square test assessed associations between categorical variables. A p-value of less than 0.05 was considered statistically significant.

Ethical approval

The Institutional Review Board (IRB) approval was obtained from the Research Ethics Review Board of the Al-Rayan National College of Medicine (approval number HA-03-M-122-039). All participants were older than 18 years and written informed consent was obtained from them before participation. The survey was available online in both English and Arabic, and validation was conducted based on previously established studies. To ensure confidentiality of participants' information in this study, all participant data were handled with strict confidentiality throughout the study. Personal identifiers were removed, and data were anonymized during data processing and analysis.

## Results

A total of 82 patients participated were enrolled in this study. Among them, 40 (48.8%) were aged between 18 and 30 years, with the majority being Saudi females (79, 96.3%). Most individuals who had undergone plastic surgery were highly educated, with 59 (72%) having attained a university education and seven (8.5%) holding post-graduate degrees. Interestingly, a significant portion of those who underwent plastic surgery were non-employees (53.7%), with 26 (31.7%) having a low income of less than SR (Saudi Riyal) 5000 as detailed in Table 1.

**Table 1:** Sociodemographic profile and data distribution of the participants (n = 82). Data are presented in frequency and percentages (n, %).

Demographical Characteristics	n	%
Age		
18-30 years	40	48.80
31-40 years	17	20.70
41-50 years	22	26.80
51 years and older	3	3.70
Gender		
Male	3	3.70
Female	79	96.30
Nationality		
Saudi	71	86.60
Non-Saudi	10	12.20
Not answered	1	1.20
Educational qualification		
Intermediate school	1	1.20
Secondary school	15	18.30
University	59	72.00
Post-graduate degree	7	8.50
Job		
Student	11	13.40
Health sector	2	2.40
Military sector	2	2.40
Private sector	10	12.20
Non-employee	44	53.70
Government sector	13	15.90
Monthly income		
Less than SR 5000	26	31.70
Between SR 5000-10,000	15	18.30
Between SR 10,000-15,000	8	9.80
More than SR 15,000	4	4.90
Not answered	29	35.40

The medical profile of participants who had undergone plastic surgery shows that 67 (81.7%) were medically healthy without significant co-morbidities. Obesity was the most common health condition, affecting 8.5% of the participants, while less frequent conditions such as diabetes, asthma, and hypertension were present in fewer than 4% of participants each. Additionally, food allergies were more common than medication allergies. Overall, most participants had no major health issues, no significant family history of inherited diseases, and minimal allergies, as outlined in Table 2.

**Table 2:** Medical profile and co-morbidities data of participants (n=82). Data are presented in frequency and percentages (n, %)

Variable	n	%
Co-morbidities		
Medically free	67	81.7
Obesity	7	8.5
Diabetes mellitus	3	3.7
Asthma	3	3.7
Hypertension	2	2.4
Other diseases	2	2.4
Is there a family history of an inherited disease?		
Yes	31	37.8
No	51	62.2
Do you have allergy?		
No	69	84.1
Yes, I have allergy from food	8	9.8
Yes, I have allergy from some medications	5	6.1

The majority (51, 62.2%) of the participants indicated that they do not have a family history of inherited diseases, while 31 (37.8%) participants reported having a family history of inherited diseases. The vast majority of participants (69, 84.1%) reported no allergies. Among those who have allergies only eight (9.8%) and five (6.1%) of the participants reported having a food allergy or medication allergies, respectively (Table 2).

**Figure 1:**
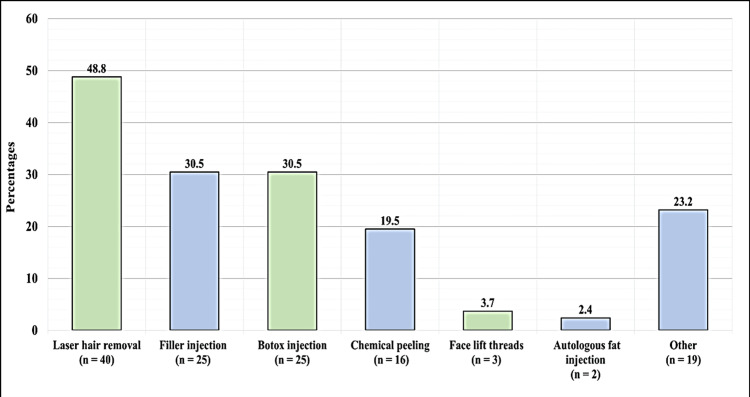
Distribution of Cosmetic Facial Procedures Among Participants, Highlighting the Popularity of Non-Invasive and Injectable Treatments (n = 82).

The most common procedure among the participants was laser hair removal 40 (48.8%), followed by filler injections and Botox injections; both were used by 25 (30.5%) of participants. Chemical peeling and procedures under the other category were moderately preferred (16 participants, 19.5%). For example, less common treatments include facelift threads and autologous fat injections. These were preferred by three (3.6%) and two (2.4%) of participants, respectively (Figure 1).

The study findings provide valuable insights into the participant’s knowledge and awareness of facial cosmetic procedures, including their understanding of the cosmetic procedures, potential complications, and the sources of their information. A significant majority (68; 82.9%) of the participants exhibited a strong general awareness. However, 23 participants (28%) indicated that they were unaware of any complications, highlighting a knowledge gap in almost one-third of the group. Furthermore, six participants (7.3%) mistakenly identified the names of cosmetic treatments, mixing up fillers and Botox, which indicated some confusion and a considerable degree of misunderstanding.

While most participants demonstrated awareness of complications related to facial cosmetic procedures, the specifics of their knowledge varied. Swelling and bruising emerged as the most recognized complications; strikingly, more severe issues such as infection and reduced facial expression were mentioned less frequently. For example, awareness of subcutaneous blood pools, infections, and decreased facial expressions was noted in 16 (19.51%), 13 (15.85%), and eight (9.76%) participants, respectively. Conversely, 53 participants (64.6%) exhibited a reasonable misunderstanding of filler injections.

It is worth noting that six participants (7.3%) mistakenly identified filler injections as botulinum toxin, reflecting some confusion between fillers and Botox. Additionally, a significant segment, 23 participants (28%) reported a lack of knowledge regarding what a filler injection entailed, highlighting a significant knowledge gap within nearly one-third of the study sample, as detailed in Table 3.

**Table 3:** Participant’s Knowledge and Awareness of Facial Cosmetic Procedures (n=82). Data are presented in frequency and percentages (n, %)

Variable	n	%
Q1: Are you aware of possible complications with any cosmetic procedure?		
Yes	68	82.9
No	14	17.1
Q2: What complications are likely to occur with facial cosmetic procedures?		
Swelling	52	63.41
Bruising	42	51.22
Rash	25	30.49
Subcutaneous blood pool	16	19.51
Infection	13	15.85
Decreased facial expressions	8	9.76
Others	4	4.88
Q3: What do you think is filler injection?		
Hyaluronic acid substance injected under the skin to fill or reduce wrinkles (correct answer)	53	64.6
A protein substance made of botulinum toxin injected into the muscles to prevent their contraction	6	7.3
I don't know	23	28
Q4: What do you think is Botox injections?		
Hyaluronic acid substance injected under the skin to fill or reduce wrinkles	16	19.5
A protein substance made of botulinum toxin injected into the muscles to prevent their contraction (correct answer)	36	43.9
Pull fatty cells from a certain area of the body	1	1.2
I don't know	29	35.4
Q5: What do you think is autologous fat injection?		
A protein substance made of botulinum toxin injected into the muscles to prevent their contraction (correct answer)	2	2.4
Pull fatty cells from a certain area of the body (correct answer)	54	65.9
I don't know	26	31.7
Q6: What do you think is the best source for information related to facial		
Medical sites	24	29.30
Health professionals	27	32.90
Family and friends’ experiences	16	19.50
Social networking sites	14	17.10
Others	1	1.20

The study findings explore the participants' (n=82) experience and attitude toward facial cosmetic procedures. Additionally, it focuses on the prevalence of complications, factors influencing decision-making, and participant’s approach to cosmetic procedures. However, the majority of participants, 51 individuals (62.2%) reported not experiencing complications after undergoing cosmetic procedures. On the contrary, a significant portion, 31 participants (37.8%) indicated that they had encountered complications.

The study results highlighted that the cost of cosmetic procedures plays a significant role in decision-making, with 62 (75.6%) of the participants stating that price affects their choice to undergo a procedure. Only 20 participants (24.4%) reported that price did not influence their decision. When selecting a doctor, the majority of the participants (43, 52.4%) prioritized the doctor's reputation, while 32 participants (39%) conducted research and consultations before making a choice. A small minority were influenced by offers and low prices (4.9%) or the clinic’s proximity (3.7%).

Regarding communication with their doctor, 32 participants (39%) reported that they always ask about the details of the cosmetic procedures and the substances used, and another 32 (39%) participants ask these questions often. However, 17 participants (20.7%) admitted they do not inquire about these details. Almost all participants (81, 98.8%) stated that they are keen to follow the doctor's advice before and after the procedure.

Remarkably, similar findings were obtained from different questions directed to the participants regarding their opinions towards planning a facelift procedure now or in the future and their planning to support people resorting to these cosmetic procedures. A similar proportion of participants (53, 64.6%) expressed their intention to support the idea of people resorting to cosmetic procedures and support people resorting to these cosmetic procedures. Conversely, 29 participants (35.4%) did not support others opting for these procedures (Table 4).

**Table 4:** Previous Experience and Attitude of participants toward Facial Cosmetic Procedures (n = 82). Data are presented in frequency (n) and percentages (%)

Variable	n	%
Previous Experience with Facial Cosmetic Procedures		
Have you had complications after a certain cosmetic procedure?		
Yes	31	37.8
No	51	62.2
Attitude toward Facial Cosmetic Procedures		
Does the price of the cosmetic procedure affect your decision to do it?		
Yes	62	75.6
No	20	24.4
What drives you to choose a specific doctor?		
The spread of the doctor's reputation	43	52.4
Choose the doctor after searching and consulting	32	39
Offers and low prices	4	4.9
The closest clinic to me	3	3.7
Do you ask your doctor about the details of the cosmetic procedure and the types and names of the substances used?		
Always	32	39
Often	32	39
I don't ask about these details	17	20.7
Not answered	1	1.2
Are you keen to follow the doctor's advice before and after the cosmetic		
Yes	81	98.8
No	1	1.2
Are you planning a facelift procedure now or in the future?		
Yes	53	64.6
No	29	35.4
Do you support people resorting to these cosmetic procedures?		
Yes	53	64.6
No	29	35.4

The study revealed the factors associated with planning a facelift procedure now or in the future and supporting other people resorting to cosmetic procedures. The findings provide a breakdown of the participant responses by age, nationality, and educational qualification. It also explores the relationship between these factors and participant’s plans for future facelifts and their support for cosmetic procedures in general. No significant associations were found with age, nationality, or education level.

When the participants were compared based on their age and nationality, a majority of participants across all age groups expressed interest in undergoing facelifts, with 62.5% of those aged 18-30, 70.6% of those aged 31-40, and 63.6% of those aged 41-50 planning for the procedure, though the p-value of 0.838 indicates no significant difference based on age. Similarly, 66.2% of the Saudi participants and 60% of the non-Saudi participants intended to have a facelift, but the p-value of 0.700 suggests that nationality does not significantly influence these intentions. Overall, age and nationality do not appear to have a substantial impact on the decision to consider facelift procedures.

Support for cosmetic procedures is generally high across all age groups, with the highest levels seen in participants aged 41-50 years (77.3%), followed by 64.7% of those aged 31-40 years, and 57.3% in the 18-30-year group. Although older participants tend to show more favorable attitudes toward these procedures, the difference in support across age groups is not statistically significant, as indicated by a p-value of 0.297.

Overall, there are some differences in facelift planning and support for cosmetic procedures across demographic groups, but none of these differences are statistically significant. Age, nationality, and educational qualification all seem to have a relatively minor impact on participant’s plans for future facelifts and their general support for cosmetic enhancements (Table 5).

**Table 5:** Factors Associated with Planning a Facelift Procedure Now or in the Future and Supporting Other People Resorting to Cosmetic Procedures. Data are presented in frequency (n) and percentages (%)

Factor	Future facelift plan		P-value
	Yes	No	
Age			0.838
18-30 years	25 (62.5%)	15 (37.5%)	
31-40 years	12 (70.6%)	5 (29.4%)	
41-50 years	14 (63.6%)	8 (36.4%)	
Nationality			0.700
Saudi	47 (66.2%)	24 (33.8%)	
Non-Saudi	6 (60%)	4 (40%)	
Educational qualification			0.359
Secondary school	8 (53.3%)	7 (46.7%)	
University	39 (66.1%)	20 (33.9%)	
Factor	Supporting people to do cosmetic procedures		P-value
	Yes	No	
Age			0.297
18-30 years	23 (57.3%)	17 (42.5%)	
31-40 years	11 (64.7%)	6 (35.3%)	
41-50 years	17 (77.3%)	5 (22.7%)	
Nationality			0.071
Saudi	49 (69%)	22 (31%)	
Non-Saudi	4 (40%)	6 (60%)	
Educational qualification			0.359
Secondary school	8 (53.3%)	7 (46.7%)	
University	39 (66.1%)	20 (33.9%)	
*Significant at level 0.05			

## Discussion

Nowadays, individuals pay significant attention to appearance as it directly impacts a person's daily life. According to Zeng et al., good appearance has a significant positive role in how much a person can get for an income. It also plays a critical role in marital satisfaction [15]. With the propaganda in all kinds of media platforms on the standards of beauty, people are constantly under pressure to uphold these standards.

In this study, the statistics has shown that the most common cosmetic facial procedures done are laser hair removal, dermal fillers, Botox injection, and chemical peeling by 48.8%, 30.5%, 30.5%, 19.5% of the participants, respectively. These results are in agreement with those reported by the American Society of Plastic Surgeons (ASPS) where laser hair removal, chemical peels, botulinum toxin type A injection, and microdermabrasion were the most common minimally invasive procedures performed [16]. In another study carried out in Singapore, researchers stated that the procedures performed by respondents were facial lesion removal, facial laser, and chemical peeling [17]. These percentages suggest that interest in facelifts is fairly consistent across age groups, with a slight peak in the 31-40-year age bracket.

Also, in this study, the vast majority (70.7%) cited that the primary motivation to undergo a cosmetic procedure was a personal desire. In contrast to this finding, Chen et al. reported that looking better in photos is the primary motivator for seeking cosmetic surgeries [18], which demonstrates the power of social media to push people into seeking cosmetic procedures. This power is driven by the fact that posting photos on social media platforms has been an integral part of socializing and getting in touch with family and friends in the past few years. Altering a person's appearance means admitting a personal imperfection, which ultimately drives the individuals to seek dermatological and surgical care [19].

Moreover, 82.9% of the participants in this study reported that they were aware of the possible complications of the cosmetic procedure, while 64.6% correctly identified that filler injection is a hyaluronic acid substance injected under the skin, and 43.9% correctly identified Botox injection as a protein substance made of botulinum toxin injected into the muscles. These results indicate the participant’s awareness level about cosmetic procedures and their complications. In a study conducted in Nigeria, 41.8% of the participants considered their awareness level toward cosmetic surgery to be low, while 22.1% considered it to be very low, which are in contrary to the findings found in this study [20]. Moreover, 51.8% of the junior college students in a Singaporean survey did not have any knowledge of the risks associated with cosmetic procedures; the study showed that 35.7% of the medical students that have participated were unaware of any risks or complications of cosmetic procedures, which indicate a relatively low level of awareness as well [17]. This discrepancy in the results between the studies could be explained by the variation in the sample size, as well as the difference in the characteristics of the targeted population. Naturally, participants with exposure to cosmetic surgeries whether academically or through personal experience are expected to have a higher level of knowledge compared to the general population.

In this investigation, participants recognized complications associated with cosmetic surgery in the following order, from highest to lowest: swelling, bruising, rash, subcutaneous blood pooling, infection, and decreased facial expressions. This suggests that while most participants had a positive experience, there remains a significant risk of adverse outcomes in cosmetic procedures. Such complications may influence participant’s attitudes and decision-making regarding future cosmetic procedures, potentially reflecting either trust in their doctors or a lack of interest in technical details.

Adejeji et al. reported that approximately 83.1% of participants acknowledged various risks associated with cosmetic surgery, including deformity, cancer, keloids, death, infection, and bleeding [20]. Another study from Nigeria identified wrinkling and disfigurement as the most common risks associated with cosmetic surgery [21]. The variation in recognition of complications across these studies may be attributed to differences in data collection methods and levels of knowledge among participants, indicating that individuals value professional guidance and take necessary precautions to achieve successful outcomes, while maintaining an ongoing interest in cosmetic enhancements.

Furthermore, 75.6% of the study participants reported that the cost of cosmetic procedures affects their decision to undergo them. In a survey conducted in India, 37% of respondents perceived plastic surgery as an expensive option reserved for the wealthy and well-known [22]. Similarly, a Nigerian study revealed that many respondents regarded cosmetic surgeries as costly, with urban areas identified as the primary locations for these procedures [21]. This indicates that affordability plays a significant role in shaping participant’s choices, potentially prompting them to explore more budget-friendly alternatives or reconsider their decision to undergo procedures altogether.

In summary, although complications from cosmetic procedures are not uncommon, most participants rely on professional advice and prioritize the doctor's reputation when making treatment decisions. Cost significantly influences their choices, and many actively seek detailed information about the procedures. A substantial proportion of participants are open to undergoing additional cosmetic procedures and generally support their use, indicating a favorable attitude toward facial cosmetic enhancements among the majority of those surveyed.

Strengths and limitations

This study has its limitations including the naturally bonded limitation of cross-sectional studies of being less accurate due to the self-administered style of data collection. Although the targeted population (adult females with a history of facial cosmetic surgeries) is considered one of the study's strengths, as it measures the level of knowledge among the group of interest, it is also considered a limitation. Having a study that targets the general population with different characteristics would generate a more generalizable finding.

## Conclusions

The majority of participants were aware of the potential complications associated with cosmetic procedures and correctly identified that filler injections involve hyaluronic acid substances injected under the skin, while Botox injections consist of a protein derived from botulinum toxin. Additionally, the study revealed that the most commonly recognized complications related to cosmetic procedures included swelling, bruising, rash, subcutaneous blood pooling, infection, and decreased facial expressions. No factors related to planning a facelift procedure were identified in this study. Educating patients who visit plastic surgery clinics about the potential complications associated with the procedures they are considering is strongly recommended. Furthermore, nationwide studies targeting the general population to explore participant’s perceptions, attitudes, and knowledge regarding facial cosmetic surgeries, as well as the social and cultural influences affecting individuals, are essential for developing a comprehensive understanding of the topic. 
